# Recent progress in research on cell-to-cell movement of rice viruses

**DOI:** 10.3389/fmicb.2014.00210

**Published:** 2014-05-20

**Authors:** Akihiro Hiraguri, Osamu Netsu, Nobumitsu Sasaki, Hiroshi Nyunoya, Takahide Sasaya

**Affiliations:** ^1^Department of Applied Biological Chemistry, Graduate School of Agricultural and Life Sciences, The University of TokyoTokyo, Japan; ^2^Department of Agricultural and Environmental Biology, Graduate School of Agricultural and Life Sciences, The University of TokyoTokyo, Japan; ^3^Gene Research Center, Tokyo University of Agriculture and TechnologyFuchu, Tokyo, Japan; ^4^Plant Disease Group, Agro-Environment Research Division, Kyushu Okinawa Agricultural Research Center, National Agriculture and Food Research OrganizationKoshi, Kumamoto, Japan

**Keywords:** cell-to-cell movement, movement protein, rice, rice virus, *trans*-complementation experiment

## Abstract

To adapt to plants as hosts, plant viruses have evolutionally needed the capacity to modify the host plasmodesmata (PD) that connect adjacent cells. Plant viruses have acquired one or more genes that encode movement proteins (MPs), which facilitate the cell-to-cell movement of infectious virus entities through PD to adjacent cells. Because of the diversity in their genome organization and in their coding sequences, rice viruses may each have a distinct cell-to-cell movement strategy. The complexity of their unusual genome organizations and replication strategies has so far hampered reverse genetic research on their genome in efforts to investigate virally encoded proteins that are involved in viral movement. However, the MP of a particular virus can complement defects in cell-to-cell movement of other distantly related or even unrelated viruses. *Trans*-complementation experiments using a combination of a movement-defective virus and viral proteins of interest to identify MPs of several rice viruses have recently been successful. In this article, we reviewed recent research that has advanced our understanding of cell-to-cell movement of rice viruses.

## INTRODUCTION

To transport their genome from an initially infected cell to neighboring cells, plant viruses need to pass through cytoplasmic channels, called plasmodesmata (PD) in rigid cell walls. Since the diameter of PD is smaller than virus particles, most plant viruses encode one or more movement proteins (MPs) that can localize at the PD and modify the structure of PD to allow passage of the virus particles into the next cells. During the processes involved in viral cell-to-cell movement, viral MPs would be associated with virus particles or viral RNA–protein complexes (vRNPs) to and through PD (Melcher, 1990; Oparka et al., 1997; Waigmann et al., 2004; Lucas, 2006; Benitez-Alfonso et al., 2010).

The first evidence suggesting that cell-to-cell movement for a certain plant virus is controlled by a viral MP was provided by a study on a 30-kDa protein of a temperature-sensitive mutant of tobacco mosaic virus (TMV) that replicates but is defective in movement at certain temperatures (Meshi et al., 1987). In reciprocal experiments using the infectious TMV mutants, wild-type TMV was blocked in the cell-to-cell movement when the TMV gene for the wild-type 30-kDa protein was replaced by the mutant protein. Furthermore, viral movement was completely lost when a frameshift mutation was introduced into the translation start codon of the gene for the 30-kDa protein. This research indicated that the 30-kDa protein is responsible for movement of the virus. These studies gave rise to similar ones on many other plant viruses, and it soon became clear that MPs are general features for both plant RNA and DNA viruses in different genera (Atabekov and Dorokhov, 1984; Waigmann et al., 2004; Lucas, 2006; Taliansky et al., 2008).

Over 15 viruses affect rice (*Oryza sativa* L.), one of the most important cereal crops for nearly half of the world’s population, and 12 are very destructive in the major rice-producing regions, especially in Asia (Hibino, 1996). These viruses are transmitted by planthoppers, leafhoppers, and chrysomelid beetles in a persistent or semi-persistent manner, or by soil-inhabiting fungus. Although the majority of plant viruses are positive-sense RNA viruses, rice viruses encompass many types of viruses, e.g., double-stranded RNA viruses [rice black-streaked dwarf virus (RBSDV) and rice dwarf virus (RDV)], segmented negative-sense RNA viruses [rice stripe virus (RSV) and rice grassy stunt virus (RGSV)], a non-segmented negative-sense RNA virus [rice transitory yellowing virus (RTYV)], a segmented positive-sense RNA viruses [rice stripe necrosis virus (RSNV) and rice necrosis mosaic virus (RNMV)], non-segmented positive-sense RNA viruses [rice yellow mottle virus (RYMV) and rice tungro spherical virus (RTSV)], and a double-stranded DNA virus [rice tungro bacilliform virus (RTBV)].

The viral MPs are involved with viral movement without affecting virus replication in single cells. In addition, even though viral MPs can be genetically swapped between different viruses, the exchangeability and complementation of movement functions have been conserved for many plant viral MPs with viruses of different families and even with plant and insect viruses (Solovyev et al., 1996; Morozov et al., 1997; Dasgupta et al., 2001; Tamai et al., 2003). On the base of these exchangeable and complementary characters of viral MPs, many virus-encoded proteins have been identified. Over the past 10 years, several uncharacterized proteins of rice viruses have been revealed to function in cell-to-cell movement (**Table 1**; Li et al., 2004; Huang et al., 2005; Xiong et al., 2008; Wu et al., 2010; Hiraguri et al., 2011, 2012; Zhang et al., 2012). In this review article, we summarized recent progress in research on cell-to-cell movement of rice viruses.

**Table 1:** Overview of movement proteins of rice viruses.

Family/genus	Virus/abbreviation	Protein/location	Notes	Reference
*Reoviridae*/*Phytoreovirus*	*Rice dwarf virus*/RDV	Pns6/Segment 6	Confirmed by complementation	Li et al. (2004)
*Reoviridae*/*Phytoreovirus*	*Rice gall dwarf virus*/RGDV	Pns7/Segment 7	Predicted by similarity to RDV MP	Moriyasu et al. (2007)
*Reoviridae*/*Fijivirus*	*Rice black-streaked dwarf virus*/RBSDV	P7-1/Segment 7	Predicted by tubular structures	Isogai et al. (1998)
*Reoviridae*/*Fijivirus*	Southern rice black-streaked dwarf virus/SRBSDV	P7-1/Segment 7	Predicted by tubular structures	Zhou et al. (2008)
*Reoviridae*/*Oryzavirus*	*Rice ragged stunt virus*/RRSV	Pns6/Segment 6	Confirmed by complementation	Wu et al. (2010)
Unassigned family/*Tenuivirus*	*Rice stripe virus*/RSV	pC4/RNA 4	Confirmed by complementation	Xiong et al. (2008)
Unassigned family/*Tenuivirus*	*Rice grassy stunt virus*/RGSV	pC6/RNA 6	Confirmed by complementation	Hiraguri et al. (2011)
Unassigned family/*Tenuivirus*	*Rice hoja blanca virus*/RHBV	pC4/RNA 4	Predicted by similarity to RSV MP	Zhang et al. (2012)
*Rhabdoviridae*/*Nucleorhabdovirus*	*Rice transitory yellowing virus*/RTYV	P3/gene 3	Confirmed by complementation	Huang et al. (2005)
Unassigned family/*Sobemovirus*	*Rice yellow mottle virus*/RYMV	P1/ORF 1 gene	Confirmed by mutagenesis	Bonneau et al. (1998)
*Caulimoviridae*/*Tungrovirus*	*Rice tungro bacilliform virus*/RTBV	Unknown/ORF 3 gene	Predicted by similarity to caulimoviral MP	Bouhida et al. (1993)
*Secoviridae*/*Waikavirus*	*Rice tungro spherical virus*/RTSV	Unknown/ORF 2 gene	Predicted by genome organization	Sanfacon et al. (2011)
Unassigned family/*Benyvirus*	*Rice stripe necrosis virus*/RSNV	TGB/RNA2	Predicted by triple gene blocks-like structure	Lozano and Morales (2009)
*Potyviridae*/*Bymovirus*	Rice necrosis mosaic virus/RNMV	P1/RNA2	Predicted by similarity to bymoviral MP	Badge et al. (1997)

## RICE-INFECTING REOVIRUSES

**FIGURE 1:**
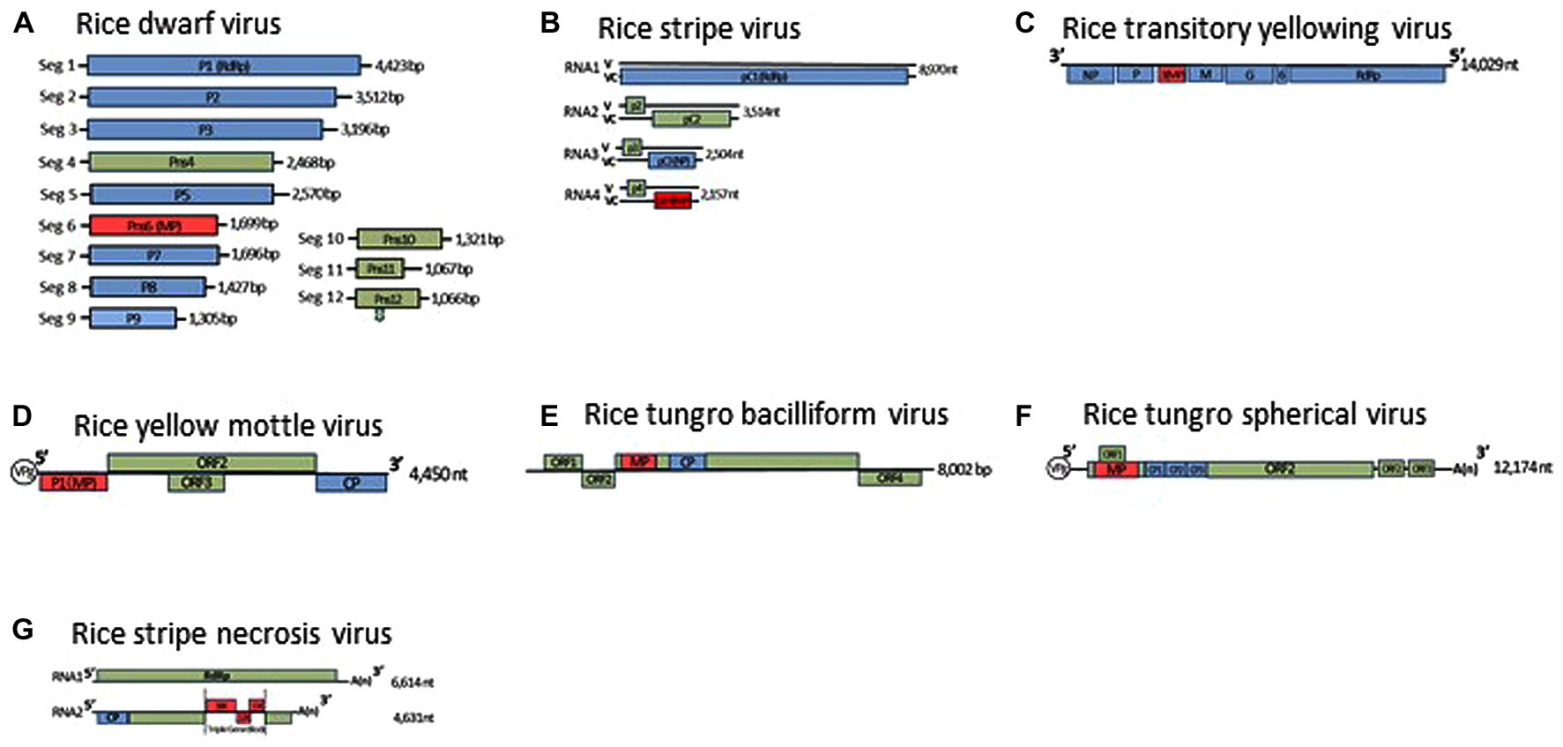
**Genome organization of rice dwarf virus **(A)**, rice stripe virus **(B)**, rice transitory yellowing virus **(C)**, rice yellow mottle virus **(D)**, rice tungro bacilliform virus **(E)**, rice tungro spherical virus **(F)**, and rice stripe necrosis virus (G).** Lines represent the viral genomic segments. Blue boxes denote genes for structural proteins, green are for non-structural proteins, red are for movement proteins (MP). CP, Coat protein; NP, nucleocapsid protein; ORF, open reading frame; RdRp, RNA-dependent RNA polymerase; V, viral-sense strand; VC, viral-complementary-sense strand.

Five reoviruses, RDV and rice gall dwarf virus (RGDV) in the genus *Phytoreovirus*, rice ragged stunt virus (RRSV) in the genus *Oryzavirus*, and RBSDV and southern rice black-streaked dwarf virus (SRBSDV) in the genus *Fijivirus*, infect rice and threaten the stability of rice production in Asia (Hibino, 1996; Hoang et al., 2011). These viruses are double-shelled spherical particles, from 50 to 80 nm in diameter, and include from 10 to 12 segmented double-stranded genomic RNAs depending on the viruses (Attoui et al., 2011; **Figure 1A**). These viruses are transmitted in a persistent manner by the insects such as *Laodelphax striatellus*, *Nilaparvata lugens*, *Nephotettix cincticeps*, *Recilia dorsalis*, and *Sogatella furcifera*, and may be replicated in both plants and in their vector insects (Hibino, 1996). RDV can be distributed in vascular bundles and in parenchymatous cells of the host plants, but the other four are localized in the phloem and the lesioned tissues of the plants. RDV is transmitted transovarially to progeny at high rates by the vector insects, but the other four are not (Hibino, 1996; Hoang et al., 2011).

To identify the MP of rice-infecting reoviruses, Li et al. (2004) used *trans*-complementation experiments in leaves of *Nicotiana benthamiana* to analyze 12 proteins encoded in the segmented RDV genome for their ability to traffic movement-defective potato virus X (PVX) mutants that were tagged with β-glucuronidase (GUS) or green fluorescent protein (GFP). The cell-to-cell movement of the mutants was restored after co-bombardment with only the plasmid containing the RDV gene for the non-structural Pns6, but not for any other RDV-encoded proteins. The complementation of viral movement was lost when the translation start codon of the gene for the Pns6 was altered from ATG to ATC. Furthermore, the GFP-fused Pns6 protein was localized near or within cell walls of epidermal cells of *Nicotiana tabacum*. Immunogold-labeling studies of thin sections from RDV-infected rice leaves using a Pns6-specific antibody showed that the Pns6 accumulated in PD of RDV-infected rice leaf cells. These results suggested that the Pns6 of RDV is the viral MP (Li et al., 2004).

Numerous studies about MPs encoded by diverse plant viruses have indicated that viral MPs have a sequence-non-specific nucleic-acid binding activity, which might represent a functional hallmark of viral MPs (Waigmann et al., 2004). The Pns6 of RDV has such sequence-non-specific binding of single- and double-stranded forms of DNAs and RNAs, but bind sequence-specifically to single-stranded forms of the viral genome, in particular, to the terminal consensus sequences of the segmented viral genome. Interestingly, the Pns6 had a stronger binding affinity to the terminal viral-sense strands than to the corresponding viral-complementary-sense strands of the RDV genome. There is a possibility that the differential binding affinity of the Pns6 may be associated with the formation of vRNPs when RDV moves through PD. In a mutagenesis analysis of Pns6, the N-terminal region of the protein was found to be responsible for the RNA-binding activities, and the conserved GKS motif, which is required for NTP binding, was also present at amino acid positions 125–127 (Ji et al., 2011). In the C-terminal region of the Pns6, the ATPase/helicase activity site, which may be involved in the unfolding of vRNPs during viral movement, was located (Lucas, 2006; Ji et al., 2011).

Pns7 of RGDV, which belongs to the same genus as RDV, is very similar to Pns6 of RDV in its amino acid sequence (Moriyasu et al., 2007). Thus, Pns7 of RGDV is assumed be functionally equivalent to Pns6 of RDV in viral movement. However, there is no direct experimental evidence to support the function of the Pns7 of RGDV as a MP.

The functioning of the non-structural Pns6 protein of RRSV in viral cell-to-cell movement has been confirmed by transient-expression experiments with the GFP-fused Pns6 protein of RRSV in epidermal cells of *Nicotiana benthamiana* and by *trans*-complementation experiments using a movement-defective TMV mutant in *Nicotiana tabacum* (Shao et al., 2004; Wu et al., 2010). Pns6 has a sequence-non-specific binding of single- and double-stranded forms of DNAs and RNAs, but binds sequence-specifically to single-stranded forms of the viral genome, and its binding domain was also determined to be located between amino acids 201 and 273 of the Pns6 of RRSV (Shao et al., 2004).

The formation of tubules that contain virus particles has been reported for many spherical viruses and considered to facilitate intercellular movement of the virus particles through the tubule structures (van Lent et al., 1991; Storms et al., 1995; Kasteel et al., 1996, 1997; Zheng et al., 1997). Similar tubular structures containing virus particles were observed in the cytoplasm of RBSDV-infected rice plants and in viruliferous vector insects (Isogai et al., 1998). Immunogold-labeled thin sections of these virus-infected rice plants and viruliferous insects indicated that the P7-1, encoded in the 5′-terminal region of RBSDV segment 7, was associated with virus-containing tubular structures (Isogai et al., 1998). Since deletion of either of two putative transmembrane domains abolishes the localization of P7-1 in the PD of *Nicotiana benthamiana* and the formation of the tubular structure in the Sf9 insect cells, these putative transmembrane domains are necessary for the P7-1 proteins to form the tubular structures (Sun et al., 2013). Thus, P7-1 is considered to function as a tubule-forming MP in both the plants and the insects, and the virus may be transported within the virus-containing tubular structures in the form of virus particles. P7-1 of SRBSDV has also been reported to function similarly in tubular formation (Zhou et al., 2008; Liu et al., 2011). However, there is no direct experimental evidence that these two proteins facilitate the cell-to-cell movement of movement-defective viruses by co-expression of these putative MPs.

## RICE-INFECTING TENUIVIRUSES

Tenuiviruses are non-enveloped, segmented, negative-sense RNA viruses. The genus presently comprises six species and six tentative species (Shirako et al., 2011). Virus particles are thin and filamentous, 3–10 nm in diameter, and composed of a single nucleocapsid protein. The viral genomes consist of four to six single-stranded RNA segments, which are either of negative polarity or of ambisense (**Figure 1B**). Since the putative RNA-dependent RNA polymerase is co-purified with viral ribonucleoproteins, the polymerase is thought to be associated with filamentous virus particles (Toriyama, 1986). Three tenuiviruses, RSV, RGSV, and rice hoja blanca virus (RHBV), were reported to cause serious problems for rice production in the world (Hibino, 1996). These viruses are transmitted in a persistent manner by the insects, such as *L. striatellus*, *Nilaparvata lugens*, and *Sogatodes orizicola*, and multiply in these insects as well as the plants. RSV and RHBV are transmitted transovarially to progeny at high rates by the vector insects, but RGSV is not (Hibino, 1996).

The RSV and RHBV genomes consist of four single-stranded RNA segments, designated RNAs 1–4 in order of decreasing molecular mass, and encode seven genes. The first RNA segment is of negative polarity, and the other three RNA segments are ambisense. The RGSV genome consists of six single-stranded RNA segments, all of which are ambisense, and includes 12 genes. The viral mRNAs are transcribed from each viral segment by the cap-snatching mechanism (Ramirez et al., 1995; Shimizu et al., 1996).

The pC4 is encoded by the viral-complementary-sense strand of RSV RNA4. Previous computer analysis of the secondary protein structures has predicted that the pC4 belongs to the “30K” superfamily of viral MPs (Melcher, 2000). As a result of co-expression of the GFP-fused pC4 protein with a PD marker protein, PDLP1a, both proteins localized together as punctate spots in cell walls (Yuan et al., 2011). An immunogold-labeling study of thin sections from the RSV-infected rice leaves using a pC4-specific antibody showed that the pC4 accumulated in cell walls of the RSV-infected rice leaves, confirming that the pC4 is a PD-localized protein (Xiong et al., 2008).

Similar to other viral MPs with sequence-non-specific binding to nucleic acids (Waigmann et al., 2004), pC4 showed sequence-non-specific single- and double-stranded RNA-binding properties in gel mobility shift assays (Xiong et al., 2008). *Trans*-complementation experiments using a GUS-tagged, movement-defective PVX mutant and later using GFP-tagged movement-defective mutants of tomato mosaic virus (ToMV) or TMV in *Nicotiana benthamiana* demonstrated repeatedly that pC4 complemented cell-to-cell movement of these movement-defective mutants, reinforcing the idea that pC4 functions as the viral MP (Xiong et al., 2008; Hiraguri et al., 2011; Zhang et al., 2012).

It is likely that RSV may be transported between plant cells in the movement form of a vRNP, rather than as a virus particle because yeast two-hybrid assays failed to show any interaction between the pC4 and the nucleocapsid protein of RSV (Xiong et al., 2008). Interestingly, inoculation of *Nicotiana benthamiana* with RNA transcripts of a TMV-pC4 hybrid, in which the pC4 gene was inserted under the CP subgenomic RNA promoter of the movement-defective TMV mutant, induced foliar necrosis in the upper leaves (Zhang et al., 2012). These results indicated that the pC4 supported not only the cell-to-cell movement but also long-distance movement of the TMV mutant. Similar results that showed the “30K” superfamily members of viral MPs support long-distance movement have been reported for several phylogenetically distinct viruses such as TMV (Knapp et al., 2001) and tomato spotted wilt virus (TSWV; Lewandowski and Adkins, 2005).

The pC4 protein of RSV requires an actomyosin motility system to access the PD via intracellular movement along the ER-to-Golgi secretory pathway (Yuan et al., 2011). Dominant-negative inhibitors were used to show that myosin VIII of the host plant cells was specifically required for pC4 localization to the PD. Myosin VIII was similarly shown to be associated with the localization of the viral MP to the PD in studies of a closteroviral MP, an Hsp70 homolog of beet yellows virus (BYV), although the same class of myosin was ineffective with respect to targeting the TMV MP, which required a myosin XI-2 to facilitate intracellular movement of TMV (Alzhanova et al., 2001; Avisar et al., 2008; Harries et al., 2009).

Although the pC4 of RSV accesses PD along the ER-to-Golgi secretory pathway, it does not have typical structural features of secreted proteins. Since pC4 lacks an N-terminal signal peptide, a transmembrane domain, and a short C-terminal tail that might direct the viral MP to the PD, intracellular movement of pC4 might involve the interaction of pC4 with host cellular proteins that are transported by Golgi-derived vesicles and are eventually anchored to the PD. With a yeast two-hybrid system using pC4, two chaperone proteins, which have high degrees of identity with a DnaJ, and a Hsp20 have been isolated from a rice cDNA library (Lu et al., 2009). Continuing research on the movement of TMV, BYV, TSWV, and potato virus Y (Soellick et al., 2000; Prokhnevsky et al., 2002; Qiu et al., 2006; Hofius et al., 2007; Shimizu et al., 2009) has shown that DnaJ proteins bind to their partner heat shock proteins of the host plant cells and seem to act as a key regulator for conformational change in the movement form of the vRNPs that allows them to pass through the PD. The heat shock proteins might have intrinsic ATPase activity and serve as motor proteins to facilitate the transport of the vRNPs through the PD. However, the actual roles of the two proteins from the rice cDNA library are unknown. The molecular mechanism by which the pC4 of RSV targets and modifies the PD for viral movement needs to be clarified.

Aside from pC4 of RSV, only the pC6 protein encoded by the viral-complementary-sense strand of RGSV RNA6, has been proven to function as a viral MP (Hiraguri et al., 2011) as shown by transient expression assays with a GFP-fused pC6 protein and a *trans*-complementation experiment with a movement-defective ToMV mutant. The functional similarities between the RSV pC4 and the RGSV pC6 are consistent with a previous prediction that RGSV RNA 6 may be functionally equivalent to RSV RNA 4 (Toriyama et al., 1997). The MP of RHBV has not yet been identified. However, the pC4 of PHBV shared a considerably high degree of similarity with the pC4 of RSV as well as the pC6 of RGSV, suggesting a common role for the pC4 orthologs in movement of tenuiviruses (Zhang et al., 2012).

## RICE-INFECTING RHABDOVIRUSES

Rice transitory yellowing virus was first identified in Taiwan in 1965 (Chiu et al., 1965) and is identical to rice yellow stunt virus (RYSV; Hiraguri et al., 2010), which was reported at almost the same time in China (Fan et al., 1965). RTYV is a member of the genus *Nucleorhabdovirus* in the family *Rhabdoviridae* and has bullet-shaped particles 180–210 nm long and 94 nm wide and has a non-segmented, negative-sense, single-stranded RNA genome (**Figure 1C**; Dietzgen et al., 2011). The virus is transmitted in a persistent manner by the insects *Nephotettix nigropictus*, *Nephotettix cincticeps*, and *Nephotettix virescens* (Chiu et al., 1965). On the basis of an SDS-PAGE analysis of the purified virus, the virus particles were first thought to consist of five proteins: nucleocapsid protein, phosphoprotein, matrix protein, glycoprotein, and RNA-dependent RNA polymerase (Hayashi and Minobe, 1985; Chiu et al., 1990; Fang et al., 1992). In recent western blot analyses and immunogold-labeling studies of the purified virus, however, the P3 and the P6 encoded by the RTYV genes 3 and 6, respectively, has also been detected in the purified virus (Huang et al., 2003; Hiraguri et al., 2012).

The genome organizations of plant-infecting rhabdoviruses are unique in having genes in addition to the basic gene orders of rhabdoviruses (Dietzgen et al., 2011). Because the gene 3, located between the genes P and M, has been identified in the genomes of all plant-infecting rhabdoviruses but not in animal-infecting rhabdoviruses (Scholthof et al., 1994; Wetzel et al., 1994; Tsai et al., 2005), the proteins encoded by gene 3 were inferred to be MPs of plant-infecting rhabdoviruses. A possible MP role for the protein encoded by gene 3 was first proposed for the sc4 protein of sonchus yellow net virus (SYNV; Scholthof et al., 1994). When the predicted secondary structure of sc4 was compared with that of known viral MPs, the sc4 was categorized in the “30K” superfamily of viral MPs (Melcher, 2000). Other proteins encoded by the gene 3 of plant-infecting rhabdoviruses, such as P3 of RYSV, 4b of lettuce necrotic yellows virus (LNYV), P3 of maize mosaic virus (MMV), and P4 of maize fine streak virus (MFSV), were also predicted to form “30K” superfamily-like secondary structures and had conserved consensus motifs homologous to the LXDX50-70G motif in the “30K” superfamily members (Huang et al., 2005).

Knowledge about the MP of plant-infecting rhabdoviruses has further advanced through the studies of the P3 protein of RTYV (synonym of RYSV). The *trans*-expressed of P3 facilitated cell-to-cell movement of a movement-defective PVX mutant in *Nicotiana benthamiana* leaves (Huang et al., 2005), and also complemented a defect in movement of a movement-defective ToMV mutant (Hiraguri et al., 2012). In transient-expression experiments with the GFP-fused P3 protein of RTYV in epidermal cells of *Nicotiana benthamiana*, P3 was associated with the nucleus and the PD. Furthermore, in immunogold-labeled thin sections of the RTYV-infected rice plants, P3 was located in the cell walls. In addition, a northwestern blot of P3 indicated that it had a single-stranded RNA-binding capacity that lacks sequence specificity *in vitro* (Huang et al., 2005). Together, these data directly suggested that P3 is a rhabdovirus MP.

An interaction between P3 and the nucleocapsid protein was also revealed in a GST pull-down assay with *E. coli*-expressed recombinant proteins of RTYV (Huang et al., 2005). On the other hand, sc4 of SYNV interacts specifically with the glycoprotein and not with the nucleocapsid protein in bimolecular fluorescence complementation experiments that examined all pairwise interactions of SYNV-encoded proteins (Min et al., 2010). In addition, the Y of potato yellow dwarf virus (PYDV), which is the MP of PYDV, interacted with the matrix protein, but not the nucleocapsid protein (Bandyopadhyay et al., 2010). Since the ribonucleocapsid is the minimal infectious unit for negative-strand viruses such as the rhabdoviruses, the sc4 and Y proteins may bind directly to the glycoprotein and matrix protein, respectively, and may associate indirectly with the nucleocapsid protein to form the viral movement complex with the nucleocapsid. In contrast, P3 of RTYV may interact directly with the nucleocapsid protein and be associated with the ribonucleocapsid core of virus particles (Hiraguri et al., 2012). The presence of P3 in the virus particles may provide an advantage for rapid viral spread to neighboring cells from the initially infected cells where the enveloped virus particles are uncoated, with subsequent release of the nucleocapsid cores. In the initial infection process of rhabdoviruses, numerous virus particles are thought to be injected into plant cells via their insect vector. Translation or replication of some virus particles may begin in the initially injected cells. Others may form an intercellular movement complex of the ribonucleocapsid immediately after they enter the neighbor cells, with the assistance of the MP that had been contained in the virus particles beforehand, and could be transported more rapidly into neighboring cells. The cell-to-cell movement strategy of the RTYV-type rhabdoviruses might be distinct from those of other enveloped plant viruses.

## OTHER RICE-INFECTING VIRUSES

### RICE YELLOW MOTTLE VIRUS

Rice yellow mottle virus is a member of the genus *Sobemovirus* (Truve and Fargette, 2011). The virus is endemic to Africa and causes serious problems for irrigated rice. The virus is polyhedral, 30 nm in diameter, and is transmitted in a semipersistent manner by a number of chrysomelid beetles including *Sesselia pussilla*, *Chaetocnema pulla*, and *Trichispa sericea* (Hibino, 1996). The RYMV genome is composed of a positive-sense, single-stranded RNA and contains four open reading frames (ORFs; **Figure 1D**; Yassi et al., 1994; Truve and Fargette, 2011). An RYMV mutant that lacks expression of the P1 protein as a result of a point mutation in the ORF1 initiation codon of RYMV ORF1, replicates efficiently in rice protoplasts, but is unable to systemically infect rice plants. In transgenic plants that express P1 in *trans*, the defective mutant virus recovered its ability for systemic infection (Bonneau et al., 1998). These results indicated that P1 is the RYMV MP. P1 also has silencing suppressor activity, and mutagenesis experiments using the RYMV infectious clone revealed that cysteine at amino acid position 95 of P1 is essential for viral cell-to-cell movement and that cysteine and phenylalanine at amino acids 64 and 88 are associated with the efficiency of the RNA silencing suppressor (Voinnet et al., 1999; Sire et al., 2008).

### TUNGRO DISEASE-ASSOCIATED VIRUSES

Tungro disease, one of the most severe virus diseases of rice, is a significant threat to rice production in South and Southeast Asia (Hibino, 1996). Tungro disease is caused by a complex of two viruses, RTBV and RTSV. RTBV is mainly responsible for symptom expression, and RTSV assists transmission of both viruses by the insects *Nephotettix virescens*, *Nephotettix nigropictus*, *Nephotettix cincticeps*, and *Recilia dorsalis* (Hibino et al., 1978; Jones et al., 1991).

Rice tungro bacilliform virus is a member of the genus *Tungrovirus* in the family *Caulimoviridae* (Geering and Hull, 2011). The virus particles are bacilliform, 100–300 nm long and 30–35 nm wide, and its genome is circular, double-stranded DNA and contains four ORFs (**Figure 1E**). ORF3 encodes the largest polyprotein, which possesses the coat protein and the analogs of a pepsin-like aspartic protease, reverse transcriptase, and RNase H1 (Rothnie et al., 1994).

The identity of the participants and understanding of the mechanism for cell-to-cell movement of caulimoviruses has progressed through studies of the P1 protein of cauliflower mosaic virus (CaMV). P1 forms tubular structures that contains the virus particles. A mutation in the CaMV gene for the P1 abolishes viral movement but does not affect viral amplification. For these reasons, P1 of CaMV has been proposed as a viral MP (Perbal et al., 1993; Thomas et al., 1993). The N-terminal 350 amino acids of the protein encoded by the RTBV ORF3 contains a region with restricted similarity to the P1 protein of CaMV (Bouhida et al., 1993). Based on this similarity with P1, the N-terminal region of the protein has also been postulated to be a MP of RTBV, but there is no direct experimental evidence that the region is associated with movement of RTBV.

Rice tungro spherical virus is the type member of the genus *Waikavirus* in the family *Secoviridae*, characterized by positive-sense, single-stranded RNA. Virus particles are polyhedral, about 30 nm in diameter and consist of three CPs (CP1, CP2, and CP3; Sanfacon et al., 2011). The viral genome is presumably attached to the genome-linked viral protein (VPg) at the 5′-terminal and is polyadenylated at the 3′-terminus (**Figure 1F**). The viral genome encodes one large ORF encoding a viral polyprotein, which contains the regions of the three CPs, the cysteine-like protease, domains for an NTP-binding protein and an RNA-dependent RNA polymerase (Shen et al., 1993). In addition, two small ORFs near the 3′-terminal of the viral genome and one small ORF that overlaps the gene for the main viral polyprotein have been identified (Shen et al., 1993; Firth and Atkins, 2008). The gene for the MP of RTSV has not been identified yet, and there is no information on the molecular mechanism for cell-to-cell movement of waikaviruses. However, the several genes for viral MPs in the family *Secoviridae* have been identified, and a comparison of the genome organization of the waikaviruses with other members of the family *Secoviridae* led to the suggestion that the region for the MP of RTSV might be located at the N-terminal region of the large viral polyprotein and be followed by three CPs (Sanfacon et al., 2011). Further experiments such as *trans*-complementation experiments using different movement-defective virus mutants or subcellular localization experiments using these putative MPs fused with fluorescent protein, are needed to investigate the molecular mechanisms of movement of RTSV.

### FUNGUS-TRANSMITTED RICE VIRUSES

Two rice viruses, RSNV and RNMV, were reported to be transmitted by a soil-inhabiting fungus, *Polymyxa graminis* (Hibino, 1996). RSNV is a tentative species of the genus *Benyvirus* (Gilmer and Ratti, 2011). The virus continues to spread in Latin America through the international trades of rice seeds produced in fields contaminated with virus-carrying fungal vectors. The virus particles have at least two rod-shaped structures that are 360 and 260 nm long, respectively, and 20 nm wide (Morales et al., 1999). The RSNV genome consists of two single-stranded RNAs, and its genome organization is nearly identical to those of beet necrotic yellow vein virus (BNYVV; **Figure 1G**; Lozano and Morales, 2009). RNA 2 of RSNV possesses three overlapping ORFs that code for polypeptides of 38.4, 12.3, and 15 kDa at amino acid positions 2194–3975, which have a typical motif of triple gene blocks (TGB). The amino acid sequences of these three overlapping ORFs of RSNV are closely related to the TGB of BNYVV, which has been revealed by site-directed mutagenesis as essential for viral movement (Gilmer et al., 1992). These polypeptides are hypothesized to be involved in movement of the virus in infected plants, but again there is no direct experimental evidence to support this hypothesis.

Rice necrosis mosaic virus is a species of the genus *Bymovirus* in the family *Potyviridae* (Adams et al., 2011). The virus particles have flexuous, filamentous structures 550 and 205 nm long and 13–14 nm wide and contain two single-stranded RNAs (Inouye and Fujii, 1977). The virus has been found in Japan and India, but is now less troublesome in these countries. RNMV is serologically related to barley yellow mosaic virus, and only the partial nucleotide sequence at the 3′-terminal of the RNMV RNA1 has so far been determined (Badge et al., 1997). By a reserve genetic system for BaYMV, the P1 protein that is encoded at the 5′-terminal of the BaYMV RNA2 has been indicated to be involved in viral movement (You and Shirako, 2010). Similarly then, the corresponding P1 encoded at the 5′-terminal of the RNMV RNA2 might also be involved in movement of RNMV. Further analyses are, however, required to determine the complete genome structure of RNMV and investigate the molecular mechanisms of its cell-to-cell movement.

## CONCLUSION AND PERSPECTIVES

Viral MPs are defined experimentally; when the proteins are mutagenized, they interfere with viral cell-to-cell movement but do not affect virus replication competence in single cells. By using reverse genetic systems for plant viruses, many virus-encoded proteins have been confirmed as viral MPs. But the complexity of the unusual genome organizations and replication strategies for many rice viruses have so far prevented the development of infectious clone systems. To date, only two infectious clone systems of RTBV and RYMV have been developed (Dasgupta et al., 1991; Bonneau et al., 1998). Lack of reverse genetic systems for the rice viruses has made it impossible to use standard mutagenesis methods for functional studies of their encoded proteins that are involved in viral cell-to-cell movement. The viral MP of one particular virus can, however, complement movement not only of closely related but also of distantly related or even unrelated viruses, in spite of the striking diversity of viral movement strategies and the lack of amino acid sequence similarities among the MPs of different virus groups (Nejidat et al., 1991; Solovyev et al., 1996; Morozov et al., 1997; Morozov and Solovyev, 2003; Tamai et al., 2003). By a different experimental approach, that is, using movement-defective viruses by *trans*-complementation experiments with virus proteins of interest, the movement functions of several uncharacterized proteins of rice viruses have been determined (**Table 1**; Li et al., 2004; Huang et al., 2005; Xiong et al., 2008; Wu et al., 2010; Hiraguri et al., 2011, 2012; Zhang et al., 2012).

One of the unique features that many rice viruses are amplified in both plant and insect cells. The Pns10 protein of RDV forms tubular structures in cells of the insect vector and is thought to be associated with intercellular movement in the insect (Wei et al., 2006, 2008; Chen et al., 2012), but the protein does not facilitate movement of the movement-defective PVX mutant in plants (Li et al., 2004). The Pns6 protein of RDV functions as a viral MP in plants, but similar tubular structures are rarely found in RDV-infected rice plants (Fukushi et al., 1962; Shikata, 1969; Boccardo and Milne, 1984). These results indicated that the strategy for intercellular movement by RDV in the insect seems to differ from that in the plant. In contrast, P7-1 of RBSDV forms tubular structures containing virus particles in the RBSDV-infected rice plants and the viruliferous insects, and the protein is considered to function as the tubule-forming MP in both the host plants and the insects (Isogai et al., 1998). The differences in the strategy for movement in plants between RDV and RBSDV may be associated with their different distributions in the plants; RDV is distributed in vascular bundles and in parenchymatous cells, and RBSDV is localized only in the plant phloem and tissues with lesions. Or these unique features of the viral MPs of rice viruses may be required specifically to infect monocotyledonous rice plants. But there is little information on the molecular details for the cell-to-cell movements of rice viruses because most of well-known MPs are encoded in the viruses that infect dicotyledonous plants. To advance research on cell-to-cell movements of rice viruses, efficient experimental methods, such as complementary systems, that work in monocotyledonous plants must be developed; most experimental methods have been developed for dicotyledonous plants and their viruses rather than for monocotyledonous plants.

Plant viruses recruit host factors that facilitate viral cell-to-cell movement through PD and influence the efficiency of viral movement. Numerous studies on the interactions between viral MPs and host factor(s) have progressively revealed the molecular mechanisms by which viral MPs target and modify PD for viral movement (Chen and Citovsky, 2003; Min et al., 2010; Ueki et al., 2010; Amari et al., 2011). Many host factors involved in viral movement have been identified from dicotyledonous plants, but not as many host factors have been identified for monocotyledonous plants (Lu et al., 2009). To advance research on cell-to-cell movement of the rice viruses, we also need to learn more about the host factors that interact with viral MPs during the PD gating process. More experimental evidence is critically needed to understand the molecular details for the cell-to-cell movement of rice viruses and to elucidate the mechanisms underlying movement strategies of viruses in rice.

## Conflict of Interest Statement

The authors declare that the research was conducted in the absence of any commercial or financial relationships that could be construed as a potential conflict of interest.
